# Community-Based Interventions as Opportunities to Increase HIV Self-Testing and Linkage to Care Among Men Who Have Sex With Men – Lessons From Ghana, West Africa

**DOI:** 10.3389/fpubh.2021.660256

**Published:** 2021-06-11

**Authors:** Gamji M'Rabiu Abubakari, DeAnne Turner, Zhao Ni, Donaldson F. Conserve, Debbie Dada, Amma Otchere, Yaw Amanfoh, Francis Boakye, Kwasi Torpey, LaRon E. Nelson

**Affiliations:** ^1^Center for Interdisciplinary Research on AIDS, School of Medicine, Yale University, New Haven, CT, United States; ^2^School of Medicine, Yale University, New Haven, CT, United States; ^3^Milken Institute School of Public Health, George Washington University, Washington, DC, United States; ^4^School of Nursing, Yale University, Orange, CA, United States; ^5^School of Social Work at Hunter College, New York, NY, United States; ^6^Priorities on Rights and Sexual Health, Accra, Ghana; ^7^Department of Population, Family, and Reproductive Health, School of Public Health, University of Ghana, Accra, Ghana; ^8^Yale Institute of Global Health, Yale University, New Haven, CT, United States; ^9^MAP Center for Urban Health Solutions, Unity Health Toronto, Toronto, ON, Canada

**Keywords:** MSM, Ghana, community-based intervention, HIV self testing, mobile health, HIV peer support

## Abstract

MSM in Ghana encounter challenges in accessing HIV services and may experience barriers to HIV self-testing due to multiple forms of stigma present in health care settings. We worked with community-based organization partners to implement three interventions that successfully engaged and retained MSM which provides an opportunity for linkage to self-testing and medical care. These interventions were (1) Many Men Many Voices (3MV) a locally-led culturally grounded group-level HIV prevention program, (2) Auntie's Corner: a mobile-app based connecting MSM to health monitoring by a registered nurse and (3) HIV Education, Empathy, & Empowerment (HIVE3): a mobile-app based peer support intervention for MSM living with HIV. The 3MV intervention may be effective in improving HIV self-testing due to its effectiveness in engaging MSM, increasing HIV testing, and improving MSM understanding of the need for HIV testing. The utilization of apps like Auntie's Corner could positively impact HIV self-testing among MSM because it increases contact with nurses and reporting of symptoms. In HIVE3, participants expressed appreciation of the security and privacy that protects their identities as MSM and the peer mentors' abilities to make referrals to the nurses in Auntie's Corners. The confidentiality component has proven key among MSM and connecting MSM to self-testing through apps to report their process and receive care could increase utilization. Together, we show the efficacy of using the community-engaged process in reaching and engaging highly stigmatized populations like Ghana and sub-Saharan Africa, and its potential in increasing HIV self-testing and linkage to HIV care.

## Introduction

Globally, men who have sex with men (MSM) have an ~26 times greater risk of contracting HIV than the general population (1). Sub-Saharan Africa (SSA) remains disproportionately affected by the global HIV epidemic– accounting for two-thirds of the global disease burden and 73% of HIV-related deaths (2). MSM in SSA countries such as Ghana carry a disproportionate burden of HIV compared to the general population (3, 4). Yet, HIV testing among MSM in SSA remains low; a large proportion (two-thirds) of MSM living with HIV in South Africa, Kenya, Malawi, and Mozambique remain unaware of their serostatus (5). Although, some SSA countries like Ghana have increased efforts to improve HIV testing by increasing testing sites, MSM still face significant accessibility barriers (6). Stigma (against MSM identity, gender expression, and HIV status) and misconceptions (e.g., low-risk perception) dissuade MSM from testing regularly (7–9). Many MSM express concerns of confidentiality, discrimination, and judgmental interactions with healthcare workers (6, 7, 10) and never tested for HIV or do not test regularly (11, 12).

HIV self-testing (HIVST) technology can increase HIV testing among MSM as it allows for testing in the privacy of their homes (13). Until recently, HIVST was mainly available in high-income countries (14, 15). The 5-year Self-Testing AfRica (STAR) Initiative facilitated a widespread scale up by generating evidence and developing strategic partnerships with manufacturers and regulators that informed the World Health Organization's decision to strongly recommend HIVST in 2016 (13, 16, 17). Currently, up to 38 countries actively implement HIVST policies, and SSA countries receive subsidies from the Gates Foundation (14–16). HIVST can dramatically increase HIV status awareness among MSM because of its acceptability, privacy, non-stigmatization, convenience, and appeal to first-time testers (18, 19). HIVST doubles HIV testing rates, causes no greater social harm than clinic-based testing, and remains associated with reduced risky sexual behavior among MSM (17, 20). While previous HIVST studies largely focused on high-income countries (18–20) recent evidence shows improved testing coverage within low-to-middle income countries, including in SSA (20–27). WHO guidelines highlight the importance of engaging community members in creating and delivering HIVST initiatives (17). However, we have not identified a community based HIVST project has been conducted among MSM in West Africa.

We have conducted three community-based HIV interventions with MSM in Ghana (Table 1) that can positively inform the implementation of HIVST programs in West Africa. These three studies include a modified version of the Many Men Many Voices – 3MV (Nyansapo) intervention, Auntie's Corner, and HIV Education, Empathy, & Empowerment (HIVE3). This paper demonstrates how HIVST implementation can be improved with community-based interventions such as the 3MV, HIVE3, and Auntie's Corner.

**Table 1:** Field lessons for self-testing studies.

**Description**	**Applications or lesson for self-testing studies**
**The efficacy of modified many men, many voices 3MV (Nyansapo) for HIV prevention among men who have sex with men in Ghana**	
Nyansapo aimed at engaging MSM through the lead of an MSM local organization to address HIV knowledge, risk, and increase positive sexual health behaviors and HIV testing. We used the ADAPT- ITT framework to modify the 3MV into a culturally acceptable Ghanaian intervention named Nyansapo. The intervention comprised seven sessions designed to reduce HIV and STI risk among MSM in Ghana. PORSH recruited 57 MSM, 56 of which participated in the four-session retreat over 60 days. They invited a nurse with expertise in STIs to contribute to the discussion. We used an explanatory mixed-method design to test the efficacy of the program. Where we collected a survey at baseline, immediately after the intervention, and one-week post-intervention. We also held focus group discussion a week post-intervention to gauge participant experiences and suggestions. We found an increase in condom use by 15% for anal sex (rel. f. = 0.80–0.95), an increase in regular HIV testing by 13%, (4–17%). Overall, each participant 1-week post-intervention reported understanding the need for HIV testing. Participants found the intervention helpful as it helped them to prepare a prevention menu that they use to self-reflect and to take conscious efforts to reduce HIV risk behaviors and engaging with HIV testing. Participants also found the retreat environment as very friendly, protected their privacy, and provided a sense of safety. As a result, they freely expressed themselves, participated in all activities, and facilitated a process of creating and maintaining social networks among MSM in the country. Details of the results are reported elsewhere [Abubakari et al. (12)].	•Nyansapo showed that MSM community-based organizations can serve as pathways for successful recruitment and retention of MSM in highly stigmatized environments for HIV self-testing. •Local MSM lead in the implementation of intervention can potentially increase utilization of self-testing as it can eliminate trust concerns and increase acceptability. •Using culturally relevant manuals can help in setting standards and processes for HIV self-testing interventions. •Nyansapo's retreat participatory format when adopted for self-testing interventions, will create a conducive and private environment for learning and demonstration of self-testing among MSM. •Considering the need for results reporting and linkage to care, self-testing interventions could consider virtual ways of communication. Participants can be part of a social network and communicate with each other and even healthcare workers *via* social media or virtual platforms to communicate challenges and also get access to services.
**Dual-intervention: nurse-led mobile app-based symptom monitoring for HIV positive MSM in Ghana (Auntie**'**s corner) and HIV education**,	
**empathy, & empowerment (HIVE3)**	
As a component of our dual intervention, Aunties Corner aimed to test the feasibility and acceptability of a smartphone-based mobile application (app) for use by HIV-positive MSM to report HIV symptoms and quality of life to registered nurses. As the second component of C5, HIVE3 aimed to connect MSM living with HIV with trained peer mentors. The goals of HIVE3 were to increase peer social support, decrease social isolation, minimize the effects of HIV and same-gender stigmas on HIV self-care and healthcare-seeking behaviors. Two local MSM organizations, PORSH), and CEPEHRG led the recruitment and implementation of Aunties Corner and HIVE3 to 61 MSM over a 60 days period. Participants received a smartphone with a pre-installed app, with notifications periodically to answer questions about HIV symptoms and the quality of their daily activities. Participants also completed a Peer Support Evaluation to rate the peer support received. For Aunties, 85 initiated contact with a registered nurse, and 97% reported their HIV symptoms. HIVE3 was also found to be feasible and acceptable among our sample of MSM living with HIV in Ghana. Most participants accessed the HIVE3 app at least one time, and about half accessed the app at least 10 times. Full results of the acceptability and feasibility study will be published elsewhere.	•Like Nyansapo, Aunties Corner, and HIVE3 showed that Partnering with organizations serving MSM stands critical to successful HIV-related programming such as HIV self-testing. •HIV self-testing studies could connect MSM with providers virtually for support and collection of self-reported data to monitor MSM's real-time HIV testing results and behaviors as MSM are comfortable in using mobile apps for sharing personal, and sensitive, health information is feasible, and acceptable. •Community-based strategies can support linkage to care after self-testing for MSM who receive a positive HIVST result. •HIVE3 showed that virtual peers can serve as a liaison between clients and nursing staff by helping to promote and provide access to testing tools and giving peer to peer guidelines. •The success of the use of peers in self-testing will be enhanced if the peers receive training to provide increase credibility. •HIVE3 showed that the success of an app-based HIV-self testing program will rely on ensuring anonymity between peers and clients.

### Nyansapo (Wisdom-knot) Overview

The modified 3MV – Nyansapo was designed in collaboration with an MSM community-based organization (CBO), Priorities on Rights and Sexual Health (PORSH) to address factors that impact HIV prevention efforts among MSM in the country. We used the ADAPT-ITT framework to modify the original 3MV intervention to create a new Nyansapo manual which was used for the implementation. The ADAPT-ITT provides a guide for needs assessment and selecting interventions and modifying the intervention to suit a new population. Nyansapo was a retreat-style intervention where participants received education on HIV and STI risks, HIV testing, and HIV preventive measures in a 3-day housed group workshop. PORSH recruited for the intervention in two stages; first by contacting clients with who they engaged in the past, secondly, using the snowball technique where participants referred others to join the program. The recruitment yielded 57 interested persons, of which 56 participated in the program held in four sessions over 60 days. The person dropped out because of ill-health at the time of the intervention. Details of 3MV and Nyansapo intervention stages and results have been published earlier (12). In brief, condom use increased by 15% for anal sex (rel. f. = 0.80–0.95), and HIV testing by 13%, (4–17%) amongst participants (12). Also, irregular testers decreased by 10% (47–37%) and 100% reported understanding the need for HIV testing (12). The intervention facilitated the preparation of a prevention menu that MSM used to identify and plan on ways to reduce HIV risk behaviors and engaging with HIV testing. The retreat environment was friendly, protected MSM privacy, and provided a sense of safety. As a result, they freely expressed themselves, participated in all activities, and created social support networks among themselves.

### HIVE3 and Aunties Corner Overview

Aunties Corner and HIVE3 were components of a secure bi-directional mobile app messaging system between MSM and a team of registered nurses and MSM peers designed to improve care coordination among MSM with structural or psychosocial barriers to accessing clinic services in Ghana. Aunties Corner linked HIV + MSM with nurses to receive services virtually and documented frequency of MSM contacts with nurses and HIV symptoms reports. HIVE3 was developed based on the Dennis Peer Support Model to connect HIV + MSM with trained peer mentors for emotional, and informational support (28). Two CBOs, PORSH, and Center for Popular Education & Human Rights Ghana (CEPEHRG), led the implementation of both interventions over 60 days with a convenience sample of 61 MSM recruited through community outreach. No dropout was recorded. In the study, each participant was issued a smartphone with a pre-installed C5 app, participants received a notification every 14 days on their C5 app reminding them of answering 20 questions about HIV symptoms and their experiences over the past 14 days. Also, participants received a notification every 30 days reminding them of answering 34 questions about the quality of their daily activities and functions over the past month. The intervention was successful in linking MSM living with HIV with care; 52 participants (85%) contacted a nurse, and 59 participants (97%) reported their HIV symptoms in the Aunties Corner. For clarity, contacting a nurse include reaching out for direct support, and reporting symptoms include just filling a survey about conditions on the app. The intervention was deemed feasible and acceptable among HIV + MSM for all indicators (supportive interactions, relationship qualities, perceived benefits, and satisfaction). Over three-quarters of the participants initiated at least one conversation with a peer. Nearly half regularly communicated with peers using the peer support app. The full results of Aunties Corner and HIVE3 studies (which were approved by Institutional Review Boards of University of Rochester in the United States, and Kwame Nkrumah University of Science and Technology in Ghana) will be published elsewhere.

## Discussion

Self-testing researchers can consider the following in ensuring reach, engagement, retention, and success in self-testing interventions among MSM in stigmatized environments.

### CBOs as Pathways for Successful Recruitment, Retention, and Implementation of HIVST

Partnering with MSM focused CBOs was critical to the success of Aunties Corner, HIVE3, and Nyansapo. The CBOs helped in recruiting MSM due to established connections with MSM and a history of providing a safe space for MSM to receive services and peer support. They also received training to lead the implementation. As such, the CBOs can distribute HIVST kits and reach MSM who avoid in-person testing sites for convenience and safety reasons (26, 29). Local CBOscan increase trust, understanding, and acceptability of the self-testing process. Hence, HIVST will be more successful if a similar approach to implementation is taken. Indeed, recruitment and HIVST distribution by peers in CBOs resulted in increased HIVST in Uganda (27) and Nigeria (26).

### Culturally Relevant Manuals Can Set Standards and Processes for HIVST Interventions

An established manual, created, reviewed, and accepted in collaboration with the CBOs can provide a standard procedure for engaging HIVST. The use of manuals helped in establishing a successful process during our implementation due to the structure it provided. It remains pertinent that the manual reflects the cultural setting and unique circumstances of the particular MSM population. As seen in the modification of the 3MV to Nyansapo, the culturally relevant contents will facilitate acceptability, relatability, and discourse that address self-testing issues relevant to the cultural setting (12).

### A Conducive and Private Environment Will Facilitate Learning and HIVST Practice

MSM face high stigma at various levels (family, friends, community) (30). Therefore, researchers engaging MSM must protect their privacy, confidentiality, and security. This practice contributed to the success of our studies and remains significant for the success of HIVST studies among MSM. In the Nyansapo, by creating a secure and private retreat environment MSM engaged freely without the threat of danger. They candidly recounted their experiences and needs (12). In the HIVE 3 and Auntie's Corners, the relative anonymity provided by the C5 app contributed to its high usage and retention rates. This appeal to anonymity was echoed in a study in Thailand where MSM who reported privacy and confidentiality concerns chose online HIVST intervention over in-person counseling and test administration supervision (31). A meta-analysis established privacy as an essential benefit of HIVST among MSM (18, 26).

### Community-Based Strategies Can Support Linkage to Care After Self-Testing for MSM Who Receive a Positive HIVST Result

We recommend that HIVST interventions should not only test but follow-up to connect HIV positive participants to care. Although, Nyansapo was successful, it failed to follow-up to continue to engage participants with testing and linkage to care. On the other hand, Our Aunties Corner, and HIVE3 virtual community-based platforms were successful at linking MSM to HIV care providers. By providing access to community support networks, the application generated trust and provided a sense of privacy and security to users, which made them feel comfortable contacting nurses on the platform. Researchers in Nigeria and China highlighted the pivotal role collaborating with CBOs played in achieving high linkage to care in HIVST studies among MSM in their countries, attaining rates of 100 and 87% respectively (26, 32). A community-based HIVST study among men in South Africa achieved a linkage rate of 68% (33). A meta-analysis on studies in SSA showed that facilitated linkage to care strategies (such as peers, community health workers, or lay counselors following-up after a positive result) increase ART initiation rates by 76% (34). As such, using innovative community-based approaches after HIVST will enhance linkage to care and reduction in viral loads among MSM living with HIV.

### Virtual Platforms Can Connect MSM With Providers and Peers for Support and Collection of Self-Reported Data to Monitor Real-Time Testing Results and Behaviors

Given the stigma associated with seeking in-person care as an MSM in Ghana or other sub-Saharan African countries (7), our findings suggest that mobile platforms and digital technology could be useful in ensuring a safe and private healthcare-seeking experience (7, 12). These findings align with prior research suggesting the benefit of digital technology in connecting MSM to health resources and care (7). Using the C4 app, Aunties Corner connected MSM with nurses trained in culturally competent care for MSM; this approach was found to be feasible and effective. Studies have also found that the use of technology for access to social networks has helped with peer support, referrals, and access to services (35). These findings are similar to those in the HIVE3 component of the C4 app. In HIVE3 we found that providing access to peers *via* an online app was a feasible and acceptable method of peer support and could be used to refer clients to medically qualified nursing staff. Although, Nyansapo did not have a virtual component, participants suggested an ongoing virtual component as a way to maintain and extend peer support after completing the Nyansapo intervention (12). A 2014 study based in Ghana found that such online social networks could even extend organization research to include MSM not already reached by the organization (35).

## Conclusion

As HIVST continues to spread across the globe and contribute immensely to increasing HIV testing acceptability among key populations, MSM within SSA countries who face extreme stigmas at various levels such as family, friends, and even from health care workers will immensely benefit from this new intervention. However, we argue HIVST interventions need to take into consideration the social circumstances facing MSM and incorporate innovative ways to reach and encourage participation among MSM in the sub-continent. Using experiences from our previous studies where we engaged MSM in Ghana (Table 1), we provide key lessons or suggestions to ensure increased acceptability and usage of HIVST among MSM in SSA. We recommend a grassroots level work that engages MSM *via* established MSM CBOs in order to increase reach, recruitment, and retention of MSM, and using MSM peer leadership in educating and providing support for MSM. Considering recent technology, we suggest the use of the internet and mobile-app technologies to engage MSM in HIVST management and support. When taken into consideration, our research lessons will help reduce physical engagement with stigmatizing environment, ensure privacy, confidentiality, and security of MSM, thereby bolstering confidence and usage of HIVST among MSM in SSA.

## Data Availability Statement

The author selected the following statement: The data analyzed in this study is subject to the following licenses/restrictions: We did not conduct a full analysis for the paper, however, we included abstracted information to provide context for yet publish results of HIVE3 and Aunties Corner. Requests to access these datasets should be directed to mohammed-rabiu.abubakari@yale.edu. And I did not detect any particular expressions.

## Ethics Statement

The studies involving human participants were reviewed and approved by Kwame Nkrumah University of Science and Technology - Ghana, and University of Rochester - United States. The patients/participants provided their written informed consent to participate in this study.

## Author Contributions

LN and GA conceptualized the paper. GA, DT, and ZN led the preparation of lessons for the 3 interventions under consideration with support from LN and DC. All other authors supported with the preparation, writing, and editing of the manuscript with GA overseeing the process and compilation of the various contributions.

## Conflict of Interest

The authors declare that the research was conducted in the absence of any commercial or financial relationships that could be construed as a potential conflict of interest.
